# Epidemiological Features of Acute Pancreatitis (AP): Largest Single-Center, Cohort Study in the Western Region of Saudi Arabia

**DOI:** 10.7759/cureus.38445

**Published:** 2023-05-02

**Authors:** Murad M Aljiffry, Mohammed F Alhazmi, Rakan Abu Alqam, Siba Z Takieddin, Moaz Abulfaraj

**Affiliations:** 1 Surgery, King Abdulaziz University Hospital, Jeddah, SAU; 2 Medicine and Surgery, Faculty of Medicine, King Abdulaziz University, Jeddah, SAU; 3 Medicine and Surgery, King Abdulaziz University Hospital, Jeddah, SAU

**Keywords:** acute pancreatitis (ap), saudi arabia, jeddah, complications, etiology, epidemiology, acute pancreatitis

## Abstract

Background: Acute pancreatitis (AP) is a medical emergency which can range in severity from a mild, self-limiting condition to a catastrophic event that results in multiorgan failure. This study aimed to evaluate the epidemiological characteristics of AP.

Methods: This study included all patients diagnosed with AP at King Abdulaziz University Hospital, a tertiary care hospital in Jeddah, Saudi Arabia, between 2017 and 2021. The main aim of this study was to investigate the frequency of AP in patients who present to the hospital with abdominal pain. Secondary objectives included analyzing the causes, complications, severity, and outcomes of the patients.

Results: A total of 67 patients were included. AP constituted 11.6% of all cases of patients presenting to the hospital with abdominal pain. Only seven patients presented with severe AP, which was significantly associated with advanced age (over 60 years old). The primary causes of AP were biliary and idiopathic pancreatitis, accounting for 80.6% of the cases. The most frequent complications observed were peripancreatic fluid collection and atelectasis, which occurred in 40.3% of cases.

Conclusion: AP is a prevalent condition in patients with abdominal pain, with biliary pancreatitis being the leading cause of the disease. The majority of patients exhibited mild to moderate severity of symptoms and experienced positive outcomes when treated appropriately.

## Introduction

Acute pancreatitis (AP) is a medical emergency characterized by inflammation of the pancreas caused by auto-digestion from pancreatic enzymes, resulting in pancreatic injury [1]. The most significant etiologies of this condition include gallstones and alcohol consumption. Other less frequent causes include idiopathic factors, iatrogenic factors, hypercalcemia, hyperlipidemia, familial predisposition, drug-induced factors, infections, and trauma [2-4]. According to recent literature in Saudi Arabia, the leading causes of the condition are gallstones and alcohol consumption, which together account for 60% of cases [2]. The clinical diagnosis of AP is made when patients present with upper abdominal pain and serum lipase and amylase levels at least three times the upper limit of normal or more [5].

Understanding the epidemiology of AP in any population is paramount in aiding diagnosis, providing optimal care, and reducing morbidity and mortality, especially given the global increase in its incidence [4]. The global annual incidence of pancreatitis in the general population ranges from 20 to 40 cases per 100,000 individuals [1, 5]. However, it is challenging to compare the global incidence with the local incidence in Saudi Arabia due to the significant lack of reported local incidence of AP and perhaps because the etiologic factors (like alcohol consumption, genetics, etc.) could be different. Two of the most recent studies, conducted in Buraydah in 2021 and Riyadh in 2019, did not report the incidence of the disease [2-3]. These two studies revealed differences in the etiology of AP and other AP-related hospitalizations, providing valuable insights into the regional variations in Saudi Arabia and their comparative analysis. However, no studies have identified the epidemiology of AP in Jeddah or the western region of Saudi Arabia.

Therefore, in this study we aim to examine the epidemiology of AP in Jeddah, the largest city in the western region of Saudi Arabia, by focusing on a tertiary care university hospital, King Abdulaziz University Hospital (KAUH). We will investigate the incidence of AP in patients who presented with abdominal pain at the emergency department of KAUH between 2017 and 2021. We will analyze demographic data, symptoms, vital signs, imaging results, etiology, severity, complications, management, length of hospital stay, and hospitalization outcomes.

## Materials and methods

Patients and databases

This retrospective cohort study was conducted at KAUH. All patients diagnosed with AP who presented to the emergency department with abdominal pain and were subsequently referred to surgical services between 2017 and 2021 were included in the study. The study excluded patients with chronic pancreatitis or those under the age of 18. Ethical approval for conducting the study was obtained from the Research Ethics Committee (REC) at King Abdulaziz University (KAU).

Definition

Acute pancreatitis (AP) can be diagnosed if at least two of the following criteria are met: (1) the patient has elevated levels of pancreatic enzymes; (2) the patient presents with abdominal pain and other symptoms consistent with AP; and/or (3) there is radiological evidence of AP, such as pancreatic enlargement, peripancreatic fat stranding or fluid accumulation, and/or pancreatic necrosis, found through CT, MRI, or ultrasonography (US).

This study identified five etiologies: biliary, alcohol-related, idiopathic, iatrogenic, and autoimmune pancreatitis. Biliary involvement is confirmed through radiological evidence of either cholelithiasis or choledocholithiasis. Alcohol-induced AP is diagnosed when there is a history of alcohol consumption and there is no other identifiable cause. Idiopathic AP is diagnosed when no other cause or origin can be identified. The diagnosis of iatrogenic AP is solely based on the patient's medical history, including a history of endoscopic retrograde cholangiopancreatography. The diagnosis of autoimmune AP is based on biochemical laboratory tests, such as immunoglobulin 4 (IgG4).

Body mass index (BMI) is a measure of body fat based on height and weight. A BMI of less than 18.5 kg/m2 is considered underweight, whereas a BMI between 18.5 kg/m2 and 24.9 kg/m2 is considered normal. A BMI between 25 kg/m2 and 29.9 kg/m2 is classified as overweight, and a BMI of 30 kg/m2 or higher is classified as obese.

Assessment of disease severity

We assessed the severity of AP using the Bedside Index for Severity of Acute Pancreatitis (BISAP), which Wu et al. introduced in 2008 [6]. We chose this index for our study because it is simple and includes commonly collected variables. The BISAP scoring system incorporates several variables, including a blood urea nitrogen (BUN) level greater than 25 mg/dL, age over 60 years, the presence of systemic inflammatory response syndrome (SIRS), detection of pleural effusion on imaging, and impaired mental status (Glasgow coma scale <15) [7] (Table 1). A BISAP score of 3 or higher is indicative of severe AP.

**Table 1:** Components of BISAP scoring system. BISAP score ranges from 0 to 5, with one point added for each variable within the first 24 h on presentation. The SIRS criteria include temperature, pulse, white blood cell count, and respiratory rate. BISAP, Bedside Index for Severity of Acute Pancreatitis; SIRS, systemic inflammatory response syndrome; BUN, blood urea nitrogen

BUN > 25 mg/dL	1
Age > 60 years	1
SIRS	1
Pleural effusion detected on imaging	1
Impaired mental statues (Glasgow coma scale <15)	1

Statistical analysis

The data were extracted using a data sheet specifically designed for the study’s objectives. All data are securely stored in an Excel spreadsheet on a protected computer to ensure patient confidentiality. The data were analyzed using the Statistical Package for the Social Sciences software. The normal data were represented using mean and standard deviation (SD). The median was utilized for non-normally distributed data. The incidence of patients diagnosed with AP who presented at the emergency room with abdominal pain or tenderness was calculated after analyzing the patients’ essential characteristics. The sample was stratified by age, gender, and etiology to facilitate comparisons of complications, management, and etiology prevalence across subgroups. The methods used for comparison were the confidence interval (CI) and the chi-squared test. A significance level of p < 0.05 was used for two-tailed tests.

## Results

Incidence of AP in KAUH

**Figure 1:**
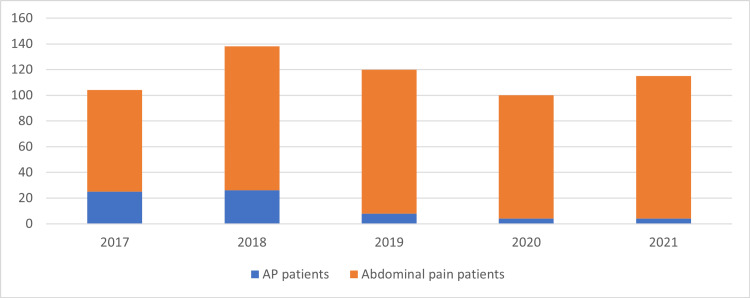
AP incidence per year. AP, acute pancreatitis

During the five-year study period, 577 patients with abdominal pain presented to the emergency room at KAUH. Among them, 67 patients were diagnosed with AP, representing 11.6% of the total referrals. In 2017 and 2018, approximately 21% of patients presenting with abdominal pain were diagnosed with AP, compared to approximately 4.6% in 2019, 2020, and 2021. There was a significant decrease in the incidence rate between 2017 and 2018 compared to 2019, 2020, and 2021 (p < 0.05) (Table 2 and Figure 1).

**Table 2:** Incidence percentage in every year. CI (95%) represents the 95% confidence interval. The total incidence between the two groups (2017 to 2018 vs 2019 to 2021) showed statistical significance (*p<0.05). AP, acute pancreatitis; ER, emergency room

Year	AP patients	ER visits for abdominal pain	Incidence (%)	CI (95%)	p-value
2017	25	104	24.0	0.1556-0.3549	
2018	26	138	18.8	0.1231-0.2761	
2019	8	120	6.6	0.0287-0.1313	
2020	4	100	4.0	0.0109-0.1024	
2021	4	117	3.4	0.0094-0.0890	<0.05*

Presentation characteristics of AP patients in the ER

During the study period, a total of 67 patients were admitted to KAUH due to AP. Of these patients, 37 (55.2%) were females and 30 (44.8%) were males. There was no significant difference in the incidence rate between genders (p > 0.05). The age of the patients ranged from 22 to 80 years (mean ± SD: 48.87 ± 15.16; median: 49), as shown in Table 3. Among the patient population, 31.3% had a BMI within the normal range, 34.3% were classified as overweight, and 32.8% were classified as obese. Only one patient was underweight, and there were no significant differences in BMI between genders. There was no significant correlation between BMI and etiology in either gender (p > 0.05). The predominant initial clinical symptom was epigastric pain, reported in 83.6% of cases. The most common associated symptom was nausea and vomiting (68.7% and 67.2%, respectively) followed by back pain (20.9%). Seven patients (10.4%) were admitted with a BISAP score of 3 or higher, indicating severe AP (see Table 4). A BISAP score of 3 or higher was found to be significantly associated with patients over 60 years old (p < 0.05). The vital signs and laboratory results of both genders are summarized in Table 3.

**Table 3:** Demographic and laboratory features of sample size.

	Minimum	Maximum	Mean	Standard deviation
Age (years)	22	80	48.8	15.1
Weight (kg)	44	175	76.1	19.1
Height (cm)	60	176	161.2	14.8
Length of stay at the hospital (days)	1	186	12.7	25.9
Length of stay at the ICU (days)	1	24	10.0	8.1
Heart rate (beats/min)	51	142	88.5	18.9
Systolic (mmHg)	69	185	133.3	22.2
Diastolic (mmHg)	36	113	75.8	14.8
Respiratory rate (breaths/min)	18	48	21.9	4.1
O2 Saturation (%)	88	100	98.2	2.2
Temperature (degree Celsius)	36	39	36.6	0.5
White blood cells (K/µL)	3	39	11.5	5.3
Hematocrit level (%)	24	52	38.2	7.0
C-reactive protein (mg/L)	3	299	103.7	98.9
Serum creatinine (umol/L)	38	710	108.5	108.8
Blood glucose level (mmol/L)	4	28	9.9	5.6
Serum amylase (U/L)	48	3359	674.4	773.1
Serum lipase (U/L)	61	30000	6786.3	8769.9

**Table 4:** Median length of stay at the hospital. Severity: BISAP score from 0 to 5, Severe: BISAP score of 3 or higher, Non-severe: BISAP score less than 3.

	Number of patients	Median length of stay at the hospital/days
Etiologies:		
Biliary	37	7
Idiopathic	17	4
Autoimmune	7	18
Alcoholic	4	3
Iatrogenic	2	10
Complications:		
Peripancreatic fluid	17	11
Atelectasis	10	7
Pancreatic pleural effusion	6	9
Pancreatic ascites	4	33
Pancreatic necrosis	3	15
Pancreatic pseudocyst	4	11
Pancreatic abscess	1	82
Chronic pancreatitis	3	41
Severity		
Severe	7	15
Non-severe	60	7
Gender		
Male	30	7
Female	37	5
Total	67	7

Etiologies of AP

**Figure 2:**
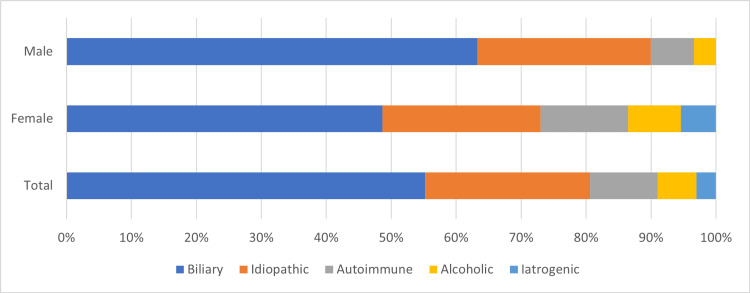
Etiological groups by gender.

Biliary pancreatitis was the most frequent cause of the condition (55.2%), and there was no significant difference in distribution between males and females (p > 0.05), as shown in Figure 2. The leading non-biliary etiology was idiopathic pancreatitis (25.4%), followed by autoimmune pancreatitis (10.4%), alcoholic pancreatitis (6%), and iatrogenic pancreatitis (3%). Out of the total number of patients at KAUH, only eight (11.9%) had a history of recurrent AP. Among the patients with recurrent AP, four had biliary pancreatitis, two had idiopathic pancreatitis, and one each had autoimmune and iatrogenic pancreatitis.

Complications of AP

Of all the patients in our study, 46.2% (mean ± SD, 31.37 ± 47.194) experienced at least one complication, whereas 53.7% (mean ± SD, 6.4 ± 5.028) did not experience any complications.

There was no statistically significant difference in the incidence of complications between males and females (p > 0.05). The most frequent complication observed was peripancreatic fluid collection (25.4%), followed by atelectasis (14.9%). Other less common complications were pancreatic pleural effusion (9%), pancreatic ascites or pancreatic pseudocysts (6%), pancreatic necrosis or chronic pancreatitis (4.5%), and pancreatic abscess (one patient).

Patients with complicated AP had a significantly longer hospital stay compared to those with noncomplicated AP (p < 0.05), as shown in Table 4. We observed that pleural effusion was significantly associated with iatrogenic AP (p < 0.05) in the group of patients who experienced complications. Patients with longer hospital stays were more likely to experience recurrent pancreatitis and develop chronic pancreatitis (mean ± SD, 49 ± 29.816; 95% CI: 8.772-67.499; p < 0.05) compared to those with the average length of stay (mean ± SD, 12.71 ± 25.945; see Table 4).

Intensive care unit (ICU) admission

Six patients (9%) were admitted to the ICU, of whom four were males. The median length of stay in the ICU was eight days (mean ± SD, 10 ± 8.173). Pancreatic necrosis was significantly associated with admission to the ICU (p < 0.05).

Types of invasive interventions

Invasive interventions were performed on 49.3% of the patients. Laparoscopic cholecystectomy was the most frequent intervention among all patients, accounting for approximately 37.3% of cases. Moreover, it was performed in approximately 65% of patients with biliary AP during the same admission. Among the patient population, 16.4% required endoscopic retrograde cholangiopancreatography, with the majority of cases being diagnosed with biliary AP (72.7%). The remaining 27.3% who underwent ERCP had non-biliary pancreatitis. Endoscopic drainage and operative debridement were performed in 4.5% of the patients. Radiological interventions, such as percutaneous drainage, were performed in 4.5% of patients. One patient required an open incision and drainage procedure with debridement.

Purpose of antibiotic therapy

Antibiotics were administered to 70% of the patients. Of those patients, 17.9% received antibiotics for treatment purposes -- specifically, for pancreatic necrosis or previously established infections -- and 52.2% received antibiotics for perioperative or postoperative prophylaxis. The most common antibiotics used for treatment purposes were cephalosporins (50%) and carbapenem (25%). For prophylactic purposes, the antibiotics used were cephalosporins (37.1%), carbapenem (28.6%), and metronidazole and tazocin (14.3%).

Imaging modality

The most frequently used imaging modality was US (59.7%), followed by CT with contrast (53.7%). The most common findings in images were fat stranding and fluid collection (41.8% and 37.3%, respectively).

## Discussion

With the paucity of studies at the national level in Saudi Arabia regarding AP, little is known regarding the epidemiology of AP. This makes it difficult to compare the incidence of AP between regions in Saudi Arabia and between Saudi Arabia and other countries. Given the importance of understanding the epidemiological features of AP in Saudi Arabia, particularly in Jeddah, we conducted a study at KAUH, a distinguished tertiary care facility. This study is the largest and only single-center investigation of AP in the western region of Saudi Arabia.

The incidence of AP among patients presenting with abdominal pain from 2017 to 2021 was 11.6%. A noteworthy decrease in the frequency of occurrence was noted when comparing the annual incidence rate between the years 2019 to 2021 with that of 2017 and 2018 (p < 0.05; see Table 2 and Figure 1). The decrease in the annual incidence rate might be attributable to the COVID-19 pandemic, which led to a significant decrease in emergency room visits and hospital admissions in Saudi Arabia [8-10] and internationally [10-11], discounting visits and admissions for infectious and respiratory diseases [12]. However, it remains uncertain whether the reduction in AP is solely attributed to the pandemic or if patients opted for self-treatment at home instead of seeking medical attention for AP.

With regard to the incidence rate of AP between genders, many Saudi researchers over the last three decades have agreed that AP is more prevalent in males than in females, except for Allhebi et al., who found no significant gender differences [2, 13-14]. However, our results, consistent with those of Allhebi et al., showed no significant difference in incidence rates between genders (p > 0.05; see Figure 2). Our findings are consistent with a study conducted in the United States, which reported no gender-based differences in the incidence of AP [15]. These results indicate that the incidence rate of AP and its association with gender may vary depending on the population under investigation and the geographical location where the study was conducted.

The BISAP score was utilized to investigate the association between AP and its severity. Our analysis revealed that the only significant correlation was with being over 60 years of age (p < 0.05). This result is consistent with the findings of Alkarawi et al. (2016), who reported that the majority of severe AP cases were observed in individuals over 55 years of age [16].

Over the course of our five-year study, we identified 67 patients with AP. Biliary pancreatitis was the most common etiology of AP, accounting for 55.2% of cases, with no significant gender disparities observed. Idiopathic pancreatitis constituted 25.4% of the cases, followed by autoimmune pancreatitis (10.4%), alcoholic pancreatitis (6%), and iatrogenic pancreatitis (3%), as shown in Figure 2. According to Saudi research, biliary pancreatitis is among the primary causes of AP [2-3]. Globally, research has also indicated that biliary pancreatitis is the primary etiology of AP [4]. In a retrospective study conducted over a period of three years and involving 37 patients, Alkhiari et al. found a similar etiology to our study regarding AP [2]. The study revealed that idiopathic (45.9%) and biliary pancreatitis (40.5%) were the two most prevalent causes. However, in a retrospective study conducted over a period of three years with 107 patients, Allehibi et al. did not identify idiopathic pancreatitis as a primary cause. Alcoholic pancreatitis was responsible for 11.2% of cases [3]. Although gallstones are the predominant cause of AP, we propose that factors contributing to AP, beyond biliary causes, may vary depending on the demographic and geographic features of the population under investigation.

Regarding the complications of AP, our study and other national studies have identified peripancreatic fluid collection as one of the most frequently occurring complications. However, in our sample we found that the second most common complication was atelectasis, a frequently reported pulmonary complication [17-18], which was not reported in either Alkhiari et al. and Allehibi et al. In contrast to previous Saudi studies reporting higher incidence rates of pancreatic pseudocysts and necrosis, our study found that both conditions were relatively uncommon, with pancreatic pseudocysts occurring in only 6% and necrosis in 4.5% of cases. This observation may be attributed to the frequent utilization of diagnostic techniques in the emergency room, including ultrasound as the primary diagnostic tool for excluding biliary etiologies of AP [19], as well as prompt surgical or endoscopic intervention that can prevent disease progression. A correlation was observed in cases of chronic pancreatitis, indicating that patients who spend longer periods in the hospital due to AP are at a higher risk of developing chronic pancreatitis in the future. This observation may be attributed to the severity of the condition, which perpetuated the destruction of the pancreatic parenchyma as a result of inflammation.

The high incidence of biliary causes of AP has led to the frequent use of laparoscopic cholecystectomy. Considered the preferred intervention for biliary pancreatitis, laparoscopic cholecystectomy is advocated for use even in mild biliary pancreatitis and as early as possible because early intervention with laparoscopic cholecystectomy has been shown to be a safe option that does not increase the risk of developing pancreatic complications [20-21].

Out of the total patients, 31.3% had a normal BMI, 34.3% were classified as overweight, 32.8% were classified as obese, and only one patient was underweight. Although we did not observe a significant association between BMI and etiology, it is worth noting that the disease appears to be more prevalent among individuals who are overweight or obese, as 67.1% of the study population had a BMI of 25 kg/m2 or higher. Nearly 60% of patients diagnosed with biliary AP and 82.3% of patients with idiopathic AP had BMIs of 25 kg/m2 or higher. A BMI of 25 kg/m2 or higher is a known risk factor for AP due to its association with cholelithiasis, hypertriglyceridemia, and diabetes, all of which increase the risk of AP [22]. Furthermore, six out of seven patients with severe AP were either overweight or obese, supporting the idea that being overweight or obese not only is a risk factor for AP but also increases its severity [22].

## Conclusions

In conclusion, a considerable proportion of patients who present at the emergency room (ER) with abdominal pain are diagnosed with AP, with an incidence rate of 11.6%. Biliary and idiopathic causes were the most frequently observed etiologies of AP. Only a small number of patients experienced severe AP. Peri-pancreatic fluid collection was the most common complication, accounting for a quarter of our sample, followed by pulmonary complications. The study was limited by its retrospective nature, and it was conducted solely in one center. We recommend further studies in a prospective manner at the national level involving multiple centers to increase patient recruitment. Our study offers a thorough analysis of the epidemiology of AP in Jeddah. This analysis is crucial for enhancing the diagnosis, care, and management of this condition. The findings of this study can be utilized to compare hospitalizations related to AP in Jeddah with those in other regions. Additionally, we suggest that future research explore the possibility of managing mild AP at home instead of in the hospital.
